# lncRNA *NAS1* Deficiency Drives Cisplatin Resistance via NR2F1-Mediated TGFB1/NF-κB Signaling Axis in NSCLC

**DOI:** 10.3390/cancers18071159

**Published:** 2026-04-03

**Authors:** Xianrong Lin, Yuxin Wu, Qi Wu, Wenjun Tao, Jun Zhang, Jun Zhou

**Affiliations:** 1 State Key Laboratory of Natural Medicines, China Pharmaceutical University, Nanjing 211198, China; 2 School of Life Science and Technology, China Pharmaceutical University, Nanjing 211198, China; 3 Jiangsu Key Laboratory of Drug Design and Optimization, China Pharmaceutical University, Nanjing 211198, China

**Keywords:** cisplatin resistance, *NAS1*, NR2F1, TGFB1, NF-κB signaling

## Abstract

**Background:** Cisplatin resistance remains a major challenge in the treatment of non-small cell lung cancer (NSCLC). Although long non-coding RNAs (lncRNAs) have been implicated in chemotherapy resistance, their specific roles and underlying mechanisms remain incompletely understood. This study aimed to identify lncRNAs associated with cisplatin resistance in NSCLC and to elucidate the role and mechanism of *NR2F1-AS1* (*NAS1*) in this process. **Methods:** Cisplatin-resistant NSCLC cell lines were established and subjected to transcriptome-wide RNA sequencing. *NAS1* expression was validated by quantitative real-time PCR, and its clinical relevance was assessed using The Cancer Genome Atlas (TCGA) dataset. *NAS1* and *NR2F1* knockdown, together with *NR2F1* overexpression and rescue experiments, were performed to evaluate their effects on cisplatin sensitivity. Downstream mechanisms were investigated by public dataset analysis, qPCR, and Western blotting, and NF-κB signaling was functionally assessed using the inhibitor DHMEQ. **Results:** *NAS1* was consistently downregulated in multiple cisplatin-resistant NSCLC cell lines and was also decreased in NSCLC tissues. *NAS1* knockdown enhanced cisplatin resistance. Mechanistically, loss of *NAS1* reduced NR2F1 protein expression without significantly affecting its mRNA level, indicating translational regulation. NR2F1 downregulation also conferred cisplatin resistance, phenocopying the effect of *NAS1* loss, whereas NR2F1 re-expression restored cisplatin sensitivity. Further analyses identified *TGFB1* as a downstream effector derepressed by loss of the *NAS1*-*NR2F1* axis, leading to activation of NF-κB signaling. Inhibition of NF-κB partially reversed cisplatin resistance in resistant cells. **Conclusions:** Collectively, our findings delineate a *NAS1*/*NR2F1*/*TGFB1*/NF-κB signaling axis critical for cisplatin resistance in NSCLC, revealing a potential therapeutic target for overcoming platinum resistance.

## Simple Summary

Cisplatin resistance is a major obstacle in the treatment of non-small cell lung cancer (NSCLC). In this study, we identified the lncRNA *NAS1* as a consistently downregulated transcript in cisplatin-resistant NSCLC cells. Mechanistically, loss of *NAS1* reduced *NR2F1* expression, increased *TGFB1*, activated NF-κB signaling, and promoted cisplatin resistance. These findings reveal a *NAS1–NR2F1–TGFB1*–NF-κB regulatory axis involved in chemoresistance and suggest potential targets for improving platinum response in NSCLC.

## 1. Introduction

Non-small cell lung cancer (NSCLC) is the most prevalent histological subtype of lung cancer, accounting for approximately 85% of all cases and representing a leading cause of global cancer-related mortality [1]. For patients with advanced or post-operative disease, platinum-based chemotherapy, predominantly with cisplatin, remains a cornerstone of first-line treatment [2,3]. However, the clinical efficacy of cisplatin is severely limited by the frequent development of intrinsic or acquired drug resistance, which constitutes a major obstacle to achieving durable therapeutic responses and improving long-term survival [4,5]. Consequently, elucidating the underlying molecular mechanisms of cisplatin resistance is imperative for developing novel strategies to overcome this barrier and improve outcomes for NSCLC patients.

Long non-coding RNAs (lncRNAs) are a class of transcripts longer than 200 nucleotides that lack protein-coding capacity. Accumulating evidence indicates their pivotal involvement in tumor biology, where they can act as drivers or suppressors of cancer initiation, progression, and metastasis [6]. Notably, their dysregulation is also increasingly linked to the development of chemotherapy resistance [7]. *NR2F1-AS1* (nuclear receptor subfamily 2 group F member 1 antisense RNA 1, hereafter referred to as *NAS1*), a recently identified lncRNA located on chromosome 5q15, has been documented in a wide array of malignancies with context-dependent regulatory functions [8]. Intriguingly, the adjacent gene *NR2F1* encodes a transcription factor reported to be translationally regulated by *NAS1* and implicated in processes such as tumor dormancy and drug resistance [9,10]. However, the precise mechanism through which *NAS1* modulates cisplatin resistance via *NR2F1* in NSCLC has not yet been fully characterized. Therefore, this study aims to determine whether the *NAS1-NR2F1* axis is involved in cisplatin resistance in NSCLC and to explore the molecular mechanisms by which its downstream factors exert their effects.

In this study, we identify that both *NAS1* and *NR2F1* are significantly downregulated in cisplatin-resistant NSCLC cell lines. Functional experiments confirm that knockdown of either gene increases cisplatin resistance, establishing their negative correlation with the cisplatin-resistant phenotype. Through rescue investigations, we demonstrate that *NR2F1* is a necessary downstream target through which *NAS1* exerts its effect. Furthermore, by integrating transcriptomic analysis and experimental validation, we reveal that downregulation of the *NAS1*-*NR2F1* axis converges on the transcriptional derepression of *TGFB1*, leading to subsequent activation of the NF-κB signaling pathway to drive cisplatin resistance. Collectively, our findings delineate a *NAS1*/*NR2F1*/*TGFB1*/NF-κB regulatory axis and suggest that targeting this pathway could represent a promising therapeutic strategy for overcoming cisplatin resistance in NSCLC.

## 2. Materials and Methods

### 2.1. Cell Culture

The human lung adenocarcinoma cell line PC9 (CellCook, CC0204, Guangzhou, China) and its cisplatin-resistant derivative PC9/CDDPr (CellCook, DR0204-DDP); the large cell lung cancer cell line H460 and its cisplatin-resistant derivative H460/CDDPr (MingJing Biology, M-C7062, Shanghai, China); as well as the lung squamous carcinoma cell line H226 (Cell Bank of Chinese Academy of Sciences, SCSP-5073, Shanghai, China) and its cisplatin-resistant counterpart H226/CDDPr were used in this study. Generated in-house by concentration-gradient intermittent induction: H226 cells were obtained from the Cell Bank of the Chinese Academy of Sciences. H226 cisplatin-resistant cells were established in-house by stepwise intermittent exposure to increasing concentrations of cisplatin over 6 months until a stable resistant phenotype was achieved. Resistant cells were maintained in medium containing 1 μM cisplatin and cultured in drug-free medium for 1 week before experiments. All cell lines were authenticated by short tandem repeat profiling within the past 3 years and routinely confirmed to be free of mycoplasma contamination. Cells were cultured in RPMI-1640 medium supplemented with 10% FBS and 1% penicillin/streptomycin at 37 °C in a humidified incubator with 5% CO_2_. All cells were cultured in RPMI-1640 medium (BI, 01-100-1ACS, Kibbutz Beit Haemek, Israel) supplemented with 10% FBS (VivaCell, C04001-500, Shanghai, China) and 1% penicillin/streptomycin (Life Technologies, 15140163, Shanghai, China). To maintain the resistant phenotype, H226/CDDPr, H460/CDDPr and PC9/CDDPr cells were routinely cultured with 1 µM, 0.83 µM and 6.6 µM cisplatin, respectively. Cells were switched to drug-free medium for one week prior to experiments. All cultures were maintained at 37 °C in a humidified atmosphere containing 5% CO_2_.

### 2.2. RNA-Seq Data Analysis

For transcriptomic profiling of cisplatin-resistant cell lines, total RNA was extracted from cells at 70–80% confluence using TRIzol reagent (Invitrogen, Carlsbad, CA, USA) according to the manufacturer’s instructions. Libraries were prepared and sequenced on an Illumina NovaSeq platform (Novogene, Beijing, China). Raw transcriptome sequencing data were first subjected to quality control using FastQC (version 0.12.1) and summarized with MultiQC [11] (version 1.33). Low-quality bases and residual adapter sequences were then removed using TrimGalore (https://github.com/FelixKrueger/TrimGalore, accessed on 7 September 2016). The cleaned reads were aligned to the human reference genome (GRCh38, Ensembl release) using Hisat2 (version 2.2.1) [12]. Gene-level read counts were quantified with FeatureCounts [13]. Differential expression analysis was performed using DESeq2 (version 1.42.1) [14]. Genes with an absolute log_2_ fold change (|log_2_FC|) > 1 and a *p*-value < 0.05 were considered significantly differentially expressed.

For the external dataset GSE233167 (gastric cancer cells with *NR2F1* knockdown or overexpression), FPKM values were downloaded from the GEO database. Genes with FPKM fold-change > 1.3 or <1/1.3 were defined as upregulated or downregulated, respectively. Heatmaps were generated for genes with |Z-score (OE_NC − KD_NC)| < 1.

### 2.3. TCGA Data Analysis

The RNA expression profiles of lung adenocarcinoma (LUAD) and lung squamous cell carcinoma (LUSC) cohorts were obtained from The Cancer Genome Atlas (TCGA) database. Primary non-small cell lung cancer (NSCLC) tumor tissues and corresponding normal lung tissues were included in the analysis. The RNA expression level of *NR2F1-AS1* was extracted from the TCGA expression datasets. Differences in *NR2F1-AS1* expression between NSCLC and normal tissues were visualized using the ggplot2 package in R (version 4.0.1). The Wilcoxon rank-sum test was applied to evaluate the statistical significance of differences in *NR2F1-AS1* RNA expression between the two groups.

### 2.4. Plasmid Construction and Transfection

To construct the sh*NR2F1* plasmid, a double-stranded oligonucleotide encoding the *NR2F1*-targeting shRNA (5′-CTCTTCTTCGTCCGTTTGGTA-3′) or a control shRNA (shScramble: 5′-AACAGTCGCGTTTGCGACTGG-3′) was cloned into the pRSI9-U6-(sh)-UbiC-TagRFP-2A-Puro vector. Correct insertion was verified by Sanger sequencing.

For *NR2F1* overexpression, the full-length human *NR2F1* coding sequence (NM_005654.6) was amplified by PCR with flanking HindIII and BamHI sites and inserted into the pcDNA3.1 vector to generate pcDNA3.1-Myc-NR2F1. Empty pcDNA3.1 was used as a negative control. Primer sequences are listed in Supplementary Table S1.

Cells were seeded in 3.5 cm dishes 24 h before transfection to reach 50–70% confluence. the pcDNA3.1 empty vector (negative control) or the pcDNA3.1-Myc-NR2F1 expression plasmid was delivered using Lipofectamine™ 3000 (Invitrogen, L3000015). Medium was replaced 4–6 h post-transfection, and subsequent experiments were conducted 24 h after transfection. Transfection efficiency was assessed 48 h after the transfection.

### 2.5. Construction of Knockdown Cell Lines

For stable *NAS1* knockdown, lentiviral particles carrying two independent shRNAs (sh*NAS1*-1: 5′-GACACTGATATAACTGTAGAT-3′; sh*NAS1*-2: 5′-GCTGCATCCTTATGGTAGCTA-3′) or a control shRNA (shNC: 5′-TTCTCCGAACGTGTCACGT-3′) were obtained from GenePharma (Shanghai, China). Cells were co-infected with both sh*NAS1* lentiviruses or infected with shNC lentiviruses at 40–50% confluence. After 24 h, the viral supernatant was removed. The cells were then cultured in complete culture medium without antibiotics for an additional 24 h. Subsequently, the cells were maintained in complete culture medium for 2 days, followed by puromycin selection (PC9: 1 µg/mL, H226: 0.5 µg/mL) was applied for 7 days to establish stable pools.

For *NR2F1* knockdown, lentivirus was produced by transfecting HEK-293T cells with packaging and sh*NR2F1* plasmids using Exfect Transfection Reagent (Vazyme, T101-02, Nanjing, China). Supernatants were collected at 24 h and 48 h, filtered (0.45 µm), and used to infect target cells at 40–50% confluence. After 24 h, complete medium was applied, and stable populations were selected with puromycin for 7 days. Knockdown efficiency was confirmed by qRT-PCR and Western blot.

### 2.6. RNA Isolation and Quantitative Real-Time PCR

Total RNA was extracted with TRIzol reagent (Invitrogen, 15596018). Reverse transcription was performed using 1 µg RNA with HiScript III RT SuperMix (Vazyme, R323-01). qPCR was carried out with ChamQ SYBR Master Mix (Vazyme, Q331-02) on a QuantStudio 3 system (Applied Biosystems, Foster City, CA, USA). Relative expression was calculated by the 2^−ΔΔCT^ method using β-actin as the internal control. All primers were synthesized by Tsingke and are listed in Supplementary Table S1.

### 2.7. Western Blot (WB)

Cells were lysed in SDS-PAGE sample buffer and heated at 100 °C for 10 min. Proteins were separated on 10% SDS-PAGE gels and transferred onto NC membranes (Sangon Biotech, F619512, Shanghai, China). Membranes were blocked with 5% non-fat milk in TBST, then incubated overnight at 4 °C with the following primary antibodies: COUP-TF1 (ab181137, Abcam, 1:1000, Cambridge, MA, USA), GAPDH (sc-365062, Santa Cruz, 1:5000, Santa Cruz, CA, USA), p-P65 (3033, CST, 1:1000, Danvers, MA, USA), P65 (8242, CST, 1:1000). After washing, membranes were incubated with HRP-conjugated anti-rabbit (SA00001-2, Proteintech, 1:5000, Wuhan, China) or anti-mouse (SA00001-1, Proteintech, 1:5000) secondary antibodies for 1 h at room temperature. Signals were detected with a ChemiDoc system (Tanon, Shanghai, China) and quantified using ImageJ (version 1.54g). All experiments were performed in triplicate.

### 2.8. Chemosensitivity Assay

Cell viability was assessed using the CCK-8 kit (Vazyme, A311-02). Cells were seeded in 96-well plates (3000 cells/well) and treated with a series of cisplatin concentrations (two-fold dilutions from 0 to 256 µM) for 48 h. Then, 10 µL of CCK-8 reagent was added per well, incubated for 1 h, and absorbance was measured at 450 nm. The half-maximal inhibitory concentration (IC_50_) was calculated by fitting a dose–response curve in GraphPad Prism 10 using the equation inhibitor vs. normalized response-variable slope.

### 2.9. Cell Migration Assay

Migration was evaluated using Transwell chambers (8 µm pore, LabSelect, 14341, Beijing, China). Cells were resuspended in medium with 1% FBS and seeded into the upper chamber (6 × 10^5^ cells/mL, 100 µL/well). The lower chamber contained medium with 10% FBS. Cisplatin (0, 4, or 8 µM) was added to both chambers. After 24 h, cells on the upper surface were removed, and migrated cells on the lower surface were fixed with 4% paraformaldehyde (Biosharp, 23355142, Beijing, China), stained with 0.1% crystal violet (Beyotime Biotechnology, C0121, Shanghai, China), imaged, and counted.

### 2.10. Colony Formation Assay

Cells were plated in 6-well plates at 700 cells/well. After adhesion, cisplatin (0, 0.5, or 1 µM) was added. Cells were cultured for 7–10 days, fixed with 4% paraformaldehyde (Biosharp, 23355142), stained with 0.1% crystal violet (Beyotime Biotechnology, C0121), and colony areas were quantified.

### 2.11. Enrichment Analysis

Gene Ontology and pathway enrichment analysis was performed using the clusterProfiler (v4.12.6) R package.

### 2.12. Statistical Analysis

All data are derived from at least three independent biological replicates. Unless otherwise specified, differences between two groups were assessed using unpaired two-tailed Student’s *t*-test. Data are presented as mean ± SEM. Significance levels are denoted as * *p* < 0.05, ** *p* < 0.01, and *** *p* < 0.001. Analyses were performed in GraphPad Prism 10.

## 3. Results

### 3.1. NAS1 Is Downregulated in Multiple Cisplatin-Resistant NSCLC Cells

**Figure 1:**
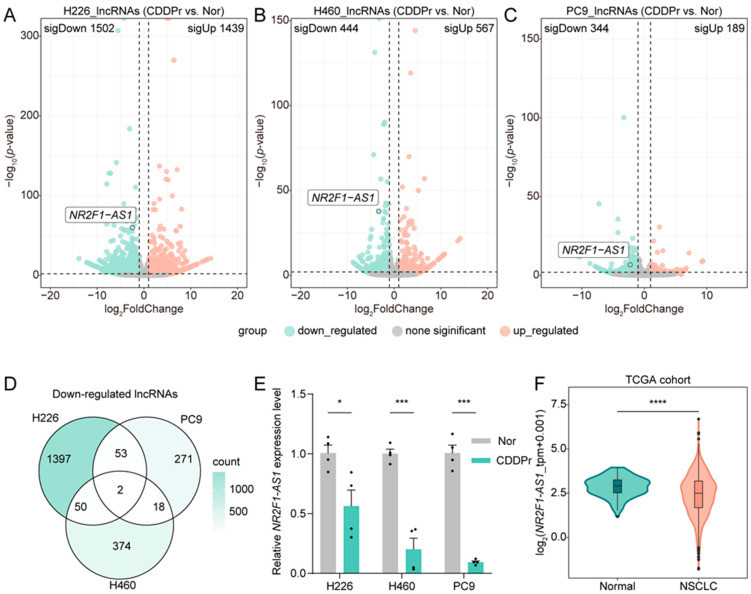
Screening and validation of *NAS1* downregulation in cisplatin-resistant NSCLC. (**A**–**C**) Volcano plots depicting differentially expressed genes between cisplatin-resistant cell lines (H226/CDDPr, H460/CDDPr, PC9/CDDPr) and their respective parental controls. Significantly upregulated and downregulated genes (threshold: |log_2_ fold change| > 1, *p*-value < 0.05) are highlighted in orange and green, respectively. (**D**) Venn diagram identifying lncRNAs consistently downregulated across all three resistant cell lines, with *NAS1* being one of the two candidates. (**E**) qPCR validation of *NAS1* expression confirms its significant downregulation in cisplatin-resistant cells compared to parental controls (n ≥ 3). (**F**) Analysis of *NAS1* expression in NSCLC patient cohorts from The Cancer Genome Atlas (TCGA) database, showing lower *NAS1* expression in tumor tissues compared to normal adjacent tissues. The data shown in the figure are presented as the mean ± SEM unless otherwise indicated. *p* values were calculated using two-tailed Student’s *t* test. * *p* < 0.05, *** *p* < 0.001, **** *p* < 0.0001.

To investigate the role of lncRNAs in cisplatin resistance of NSCLC, we performed transcriptome-wide RNA sequencing to identify differentially expressed lncRNA between three distinct cisplatin-resistant NSCLC cell lines (H226/CDDPr, H460/CDDPr, PC9/CDDPr) and their corresponding parental cells (Figure 1A–C). Widespread dysregulation of lncRNA expression was observed across all resistant cell lines. Notably, H226-resistant cells exhibited thousands of differentially expressed genes, whereas PC9-resistant cells showed a comparatively limited transcriptional response. Analysis revealed two consistently downregulated and eight consistently upregulated lncRNAs across all three resistant cell lines (Figure 1D and Figure S1A). Subsequent validation by quantitative PCR (qPCR) confirmed that only *NAS1* was significantly downregulated in all three cisplatin-resistant NSCLC models (Figure 1E and Figure S1B–E). Therefore, we further analyzed The Cancer Genome Atlas (TCGA) NSCLC cohorts and found that *NAS1* expression was consistently low in NSCLC (Figure 1F). These findings suggest that *NAS1* may play an important role in regulating cisplatin resistance in NSCLC.

### 3.2. NAS1 Knockdown Increases Cisplatin Resistance in NSCLC Cells

**Figure 2:**
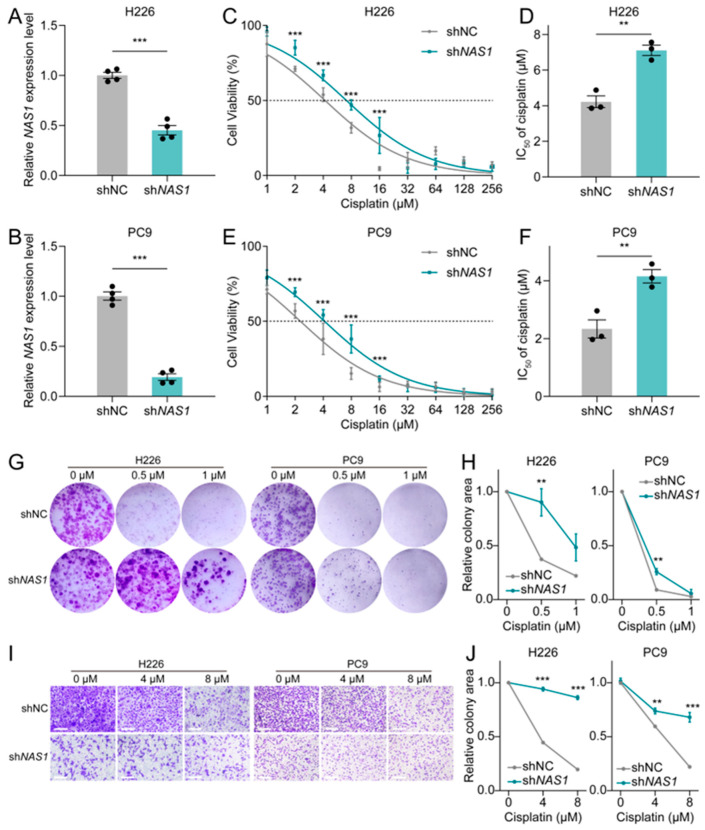
*NAS1* knockdown enhances cisplatin resistance in NSCLC cells. (**A**,**B**) Validation of *NAS1* knockdown efficiency in H226 (**A**) and PC9 (**B**) cells co-transfected with a combination of two independent shRNAs (sh*NAS1*#1 + sh*NAS1*#2) or a control shRNA (shCtrl), as measured by qPCR (n ≥ 3). (**C**–**F**) Cisplatin sensitivity measured by IC_50_ in H226 (**C**,**D**) and PC9 (**E**,**F**) cells following *NAS1* knockdown (n = 3). (**G**,**H**) Representative images (**G**) and quantification (**H**) of colony formation assays in H226 and PC9 cells treated with cisplatin (0, 0.5, or 1 μM) following *NAS1* knockdown. (**I**,**J**) Representative images (**I**) and quantification (**J**) of trans-well migration assays in H226 and PC9 cells under cisplatin treatment (0, 4, or 8 μM) after *NAS1* knockdown. Scale bar, 20 µm. The data shown in the figure are presented as the mean ± SEM unless otherwise indicated. *p* values were calculated using two-tailed Student’s *t* test. ** *p* < 0.01, *** *p* < 0.001.

To validate the negative correlation between *NAS1* expression and cisplatin resistance, we knocked down *NAS1* in H226 and PC9 cell lines by co-transfecting two independent shRNAs targeting different regions of the *NAS1* transcript (Figure 2A,B). Evaluation of the effect of *NAS1* knockdown on cisplatin sensitivity showed that loss of *NAS1* led to a roughly 2-fold elevation in the IC_50_ of cisplatin in both cell lines examined (Figure 2C–F). Moreover, *NAS1* depletion markedly attenuated the inhibitory effects of cisplatin on colony formation and cell migration (Figure 2G–J). Taken together, these findings implicate that downregulation of *NAS1* promotes cisplatin resistance in NSCLC.

### 3.3. The NAS1-Translational Regulated Gene NR2F1 Confers Cisplatin Resistance to NSCLC Cells upon Its Downregulation

**Figure 3:**
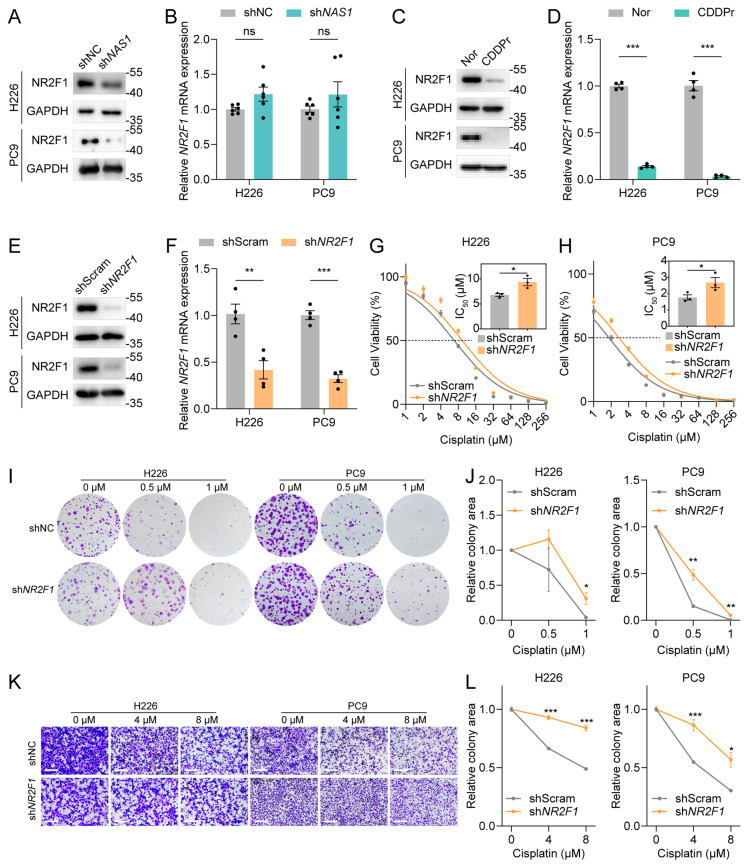
*NR2F1* acts as a downstream effector of *NAS1* to mediate cisplatin resistance. (**A**,**B**) *NAS1* knockdown reduces NR2F1 protein expression without affecting its mRNA levels in H226 and PC9 cells, as determined by Western blot (**A**) and qPCR (**B**) (n ≥ 3), confirming translational regulation. (**C**,**D**) Both *NR2F1* mRNA (**C**) and NR2F1 protein (**D**) (n ≥ 3) levels are downregulated in cisplatin-resistant cell lines compared to their parental controls. (**E**,**F**) Validation of *NR2F1* knockdown efficiency in H226 and PC9 cells transfected with *NR2F1* shRNA (sh*NR2F1*) or control shRNA (shScramble), measured by qPCR (**E**) and Western blot (**F**) (n ≥ 3). (**G**,**H**) Cisplatin sensitivity measured by IC_50_ in H226 (**G**) and PC9 (**H**) cells following *NR2F1* knockdown. (**I**,**J**) Representative images (**I**) and quantification (**J**) of colony formation assays in H226 and PC9 cells treated with cisplatin (0, 0.5, or 1 μM) following *NR2F1* knockdown. (**K**,**L**) Representative images (**K**) and quantification (**L**) of trans-well migration assays in H226 and PC9 cells under cisplatin (0, 4, or 8 μM) treatment after *NR2F1* knockdown. Scale bar, 20 µm. The data shown in the figure are presented as the mean ± SEM unless otherwise indicated. *p* values were calculated using two-tailed Student’s *t* test. ns, *p* ≥ 0.05, * *p* < 0.05, ** *p* < 0.01, *** *p* < 0.001. The uncropped blots are shown in File S1.

To elucidate the molecular mechanism by which *NAS1* regulates cisplatin resistance, we focused on *NR2F1*, a gene located adjacent to *NAS1* that encodes a transcription factor previously reported to be translationally regulated by *NAS1* and implicated in drug resistance [9,10]. To assess whether *NAS1* mediates cisplatin resistance through *NR2F1*, we first confirmed their regulatory relationship. Consistent with prior studies, *NAS1* knockdown reduced NR2F1 protein levels without affecting its mRNA expression (Figure 3A,B). Notably, both *NR2F1* mRNA and NR2F1 protein levels were downregulated in cisplatin-resistant cells (Figure 3C,D), suggesting that reduced *NR2F1* expression may contribute to cisplatin resistance. We then knocked down *NR2F1* (Figure 3E,F) and evaluated its effect on drug sensitivity. Similar to *NAS1* knockdown, *NR2F1* depletion significantly increased the IC_50_ of cisplatin (Figure 3G,H) and attenuated cisplatin-induced suppression of colony formation and cell migration (Figure 3I–L). These findings indicate that *NR2F1* downregulation promotes cisplatin resistance and that *NR2F1* appears to act as a downstream target of *NAS1* in mediating the resistant phenotype.

### 3.4. Overexpression of NR2F1 Alleviates Cisplatin Resistance Caused by NAS1 Knockdown

**Figure 4:**
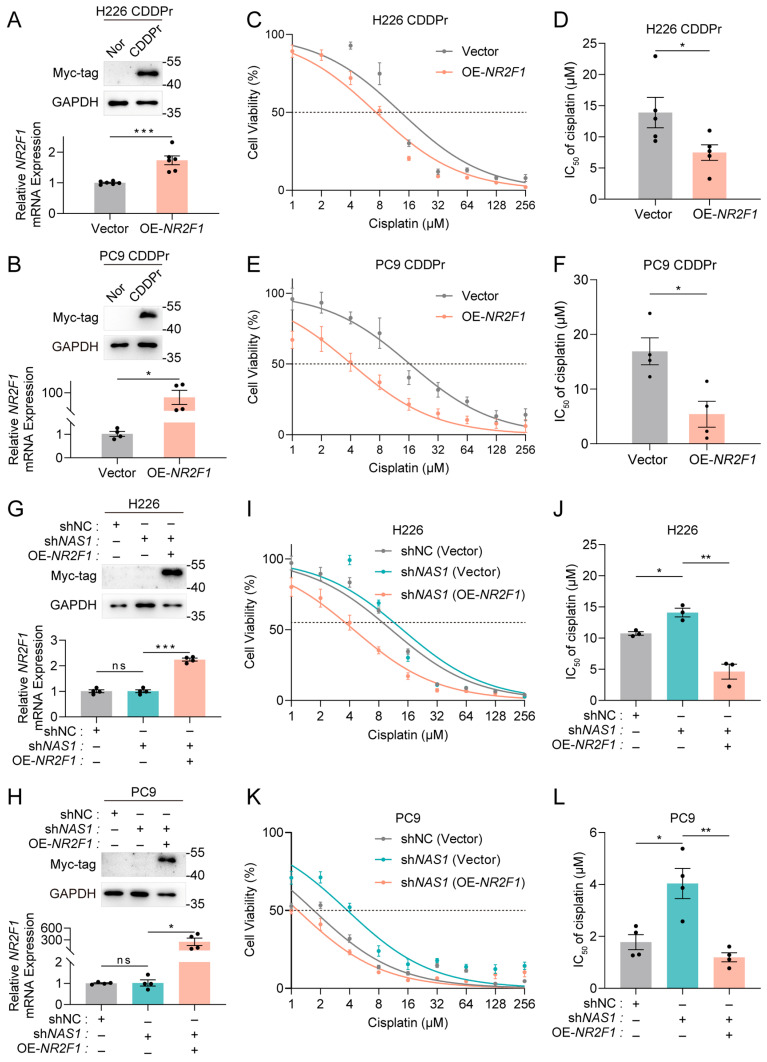
Rescue of cisplatin resistance by *NR2F1* overexpression. (**A**,**B**) Validation of *NR2F1* overexpression in cisplatin-resistant H226/CDDPr (**A**) and PC9/CDDPr (**B**) cells by Western blot (top) and qPCR (bottom, n ≥ 3). (**C**–**F**) Cisplatin sensitivity measured by IC_50_ in H226/CDDPr (**C**,**D**) and PC9/CDDPr (**E**,**F**) cells with or without *NR2F1* overexpression (n ≥ 3). (**G**,**H**) Validation of *NR2F1* re-expression in *NAS1*-knockdown H226 (**G**) and PC9 (**H**) cells by Western blot (top) and qPCR (bottom). Cells were first transfected with control (shCtrl) or *NAS1*-targeting shRNA (sh*NAS1*), followed by empty vector (Vec) or *NR2F1* expression vector (*NR2F1*-OE) (n ≥ 3). (**I**–**L**) Cisplatin sensitivity measured by IC_50_ in *NAS1*-knockdown H226 (**I**,**J**) and PC9 (**K**,**L**) cells with or without concomitant *NR2F1* re-expression (n ≥ 3). The data shown in the figure are presented as the mean ± SEM unless otherwise indicated. *p* values were calculated using two-tailed Student’s *t* test. ns, *p* ≥ 0.05, * *p* < 0.05, ** *p* < 0.01, *** *p* < 0.001. The uncropped blots are shown in File S1.

To further verify whether *NR2F1* is required for *NAS1*-mediated cisplatin resistance, we performed a rescue experiment by overexpressing *NR2F1* in two cisplatin-resistant cell lines (in which both *NAS1* and *NR2F1* are downregulated) and evaluated its impact on drug sensitivity (Figure 4A,B). The results showed that NR2F1 overexpression significantly reduced the IC_50_ of cisplatin in both resistant cell lines (Figure 4C–F). In H226-CDDPr cells, the IC_50_ decreased from approximately 13.91 μM to 7.49 μM, representing a nearly 2-fold reduction. In PC9-CDDPr cells, a more pronounced decrease was observed, with the IC_50_ dropping from 16.92 μM to 5.42 μM. These findings indicate that restoring NR2F1 expression effectively sensitizes cisplatin-resistant cells to treatment.

Moreover, restoring NR2F1 expression effectively rescued the cisplatin resistance induced by *NAS1* knockdown (Figure 4G–L). In H226 cells, NR2F1 restoration lowered the IC_50_ from 14.1 μM to 4.63 μM, a reduction of approximately 67.16%. In PC9 cells, the IC_50_ decreased even more sharply, from 4.04 μM to 1.19 μM, corresponding to a 70.54% reduction. Collectively, these findings demonstrate that *NR2F1* is a functional downstream target of *NAS1* and is necessary for mediating cisplatin resistance in NSCLC.

### 3.5. NR2F1 Regulates Cisplatin Resistance in NSCLC Through the Transcriptional Repression of TGFB1

**Figure 5:**
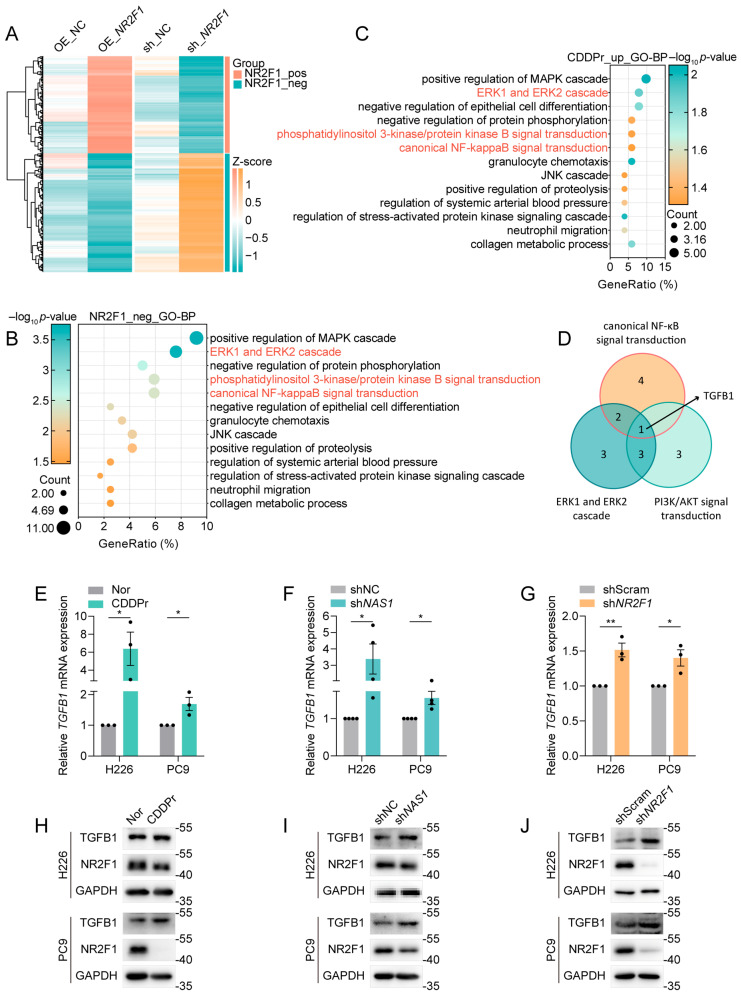
*TGFB1* is identified as a downstream effector of the *NAS1*-*NR2F1* axis. (**A**) Heatmaps showing differentially expressed genes upon *NR2F1* overexpression (OE_*NR2F1*) or knockdown (sh_*NR2F1*) in gastric cancer cells (RNA-seq dataset GSE233167). Genes upregulated upon *NR2F1* knockdown and downregulated upon *NR2F1* overexpression are defined as *NR2F1*-repressed genes (*NR2F1*-neg); genes showing the opposite pattern are defined as *NR2F1*-induced genes (*NR2F1*-pos). (**B**) GO enrichment analysis of *NR2F1*-repressed genes (*NR2F1*-neg), showing significant enrichment in drug resistance-associated signaling pathways (shown in red). (**C**) GO enrichment analysis of genes upregulated in cisplatin-resistant NSCLC cell lines compared to parental controls, revealing overlapping pathway enrichment with *NR2F1*-repressed genes. Drug resistance-associated signaling pathways are colored in red. (**D**) Venn diagram identifying *TGFB1* as the only *NR2F1*-repressed gene common to three cisplatin resistance-related pathways: NF-κB, PI3K/AKT, and ERK1/ERK2 cascade. (**E**–**G**) qPCR validation of *TGFB1* expression (n ≥ 3). *TGFB1* is upregulated in (**E**) cisplatin-resistant NSCLC cell lines, (**F**) *NAS1*-knockdown cells, and (**G**) *NR2F1*-knockdown cells, compared to their respective controls. (**H**–**J**) Western blot validation of NR2F1 and TGFB1 protein expression in H226 and PC9 cells. Consistent with the qPCR results, (**H**) cisplatin-resistant, (**I**) *NAS1*-knockdown, (**J**) and *NR2F1*-knockdown cells show reduced NR2F1 and increased TGFB1 protein levels relative to their respective controls. The data shown in the figure are presented as the mean ± SEM unless otherwise indicated. *p* values were calculated using two-tailed Student’s *t* test. * *p* < 0.05, ** *p* < 0.01. The uncropped blots are shown in File S1.

To elucidate the downstream molecular mechanisms by which the *NAS1*-*NR2F1* axis regulates cisplatin resistance, we analyzed RNA-seq datasets (GSE233167) from gastric cancer cells following *NR2F1* knockdown or overexpression. Given that the transcription factor NR2F1 has been reported to act as a transcriptional repressor of ΔNp63 [9], we focused on genes negatively regulated by NR2F1 (i.e., upregulated upon *NR2F1* knockdown and downregulated upon *NR2F1* overexpression) (Figure 5A). GO enrichment analysis revealed that these *NR2F1*-repressed genes were enriched in multiple drug resistance-associated signaling pathways (Figure 5B), which were similarly enriched among genes upregulated in cisplatin-resistant cells (Figure 5C). Among these pathways, NF-κB signaling, PI3K/AKT signaling, and the ERK1/ERK2 cascade have previously been linked to cisplatin resistance upon activation [4,15]. Notably, transforming growth factor-β1 (TGFβ1, TGFB1) was the only *NR2F1*-repressed gene involved in all three pathways (Figure 5D) and has been reported to promote drug resistance in various cancers [16,17,18]. These findings suggested that TGFB1 may be a key downstream target of the *NAS1*-*NR2F1* axis. Subsequent qPCR validation confirmed that *TGFB1* expression was upregulated in cisplatin-resistant NSCLC models as well as in *NAS1*- and *NR2F1*-knockdown cells (Figure 5E–G). Western blot analysis further confirmed the protein-level changes in NR2F1 and TGFB1 in these models. Consistent with the transcriptional results, cisplatin-resistant, *NAS1*-knockdown, and *NR2F1*-knockdown H226 and PC9 cells showed reduced NR2F1 protein expression accompanied by increased TGFB1 protein levels (Figure 5H–J). Together, these results demonstrate that *TGFB1* acts as a downstream effector of the *NAS1*-*NR2F1* axis and confers cisplatin resistance through the regulation of multiple signaling pathways.

### 3.6. Activation of the NF-κB Pathway by the Downregulated NAS1-NR2F1 Axis Drives Cisplatin Resistance

**Figure 6:**
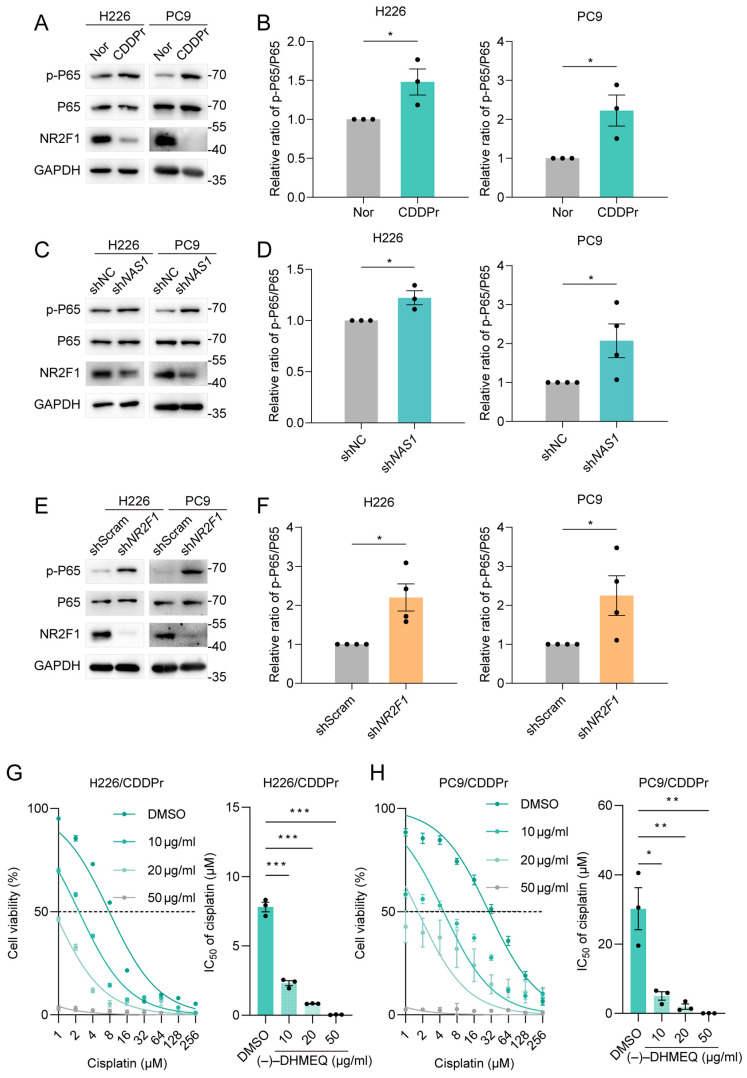
NF-κB pathway activation via downregulation of the *NAS1*-*NR2F1* axis promotes cisplatin resistance. (**A**,**B**) Elevated NF-κB activity in cisplatin-resistant NSCLC cell lines. (**A**) Representative Western blot and (**B**) quantification showing increased phosphorylation of p65 (p-p65) in H226/CDDPr and PC9/CDDPr cells compared to their parental controls (n ≥ 3). (**C**,**D**) NF-κB activation following *NAS1* knockdown. (**C**) Representative Western blot and (**D**) quantification of p-p65 levels in H226 and PC9 cells transfected with control (shNC) or *NAS1*-targeting shRNA (sh*NAS1*) (n ≥ 3). Data are presented as mean ± SEM; * *p* < 0.05 (two-tailed Student’s *t*-test). (**E**,**F**) NF-κB activation following *NR2F1* knockdown. (**E**) Representative Western blot and (**F**) quantification of p-p65 levels in H226 and PC9 cells transfected with control (shScramble) or *NR2F1*-targeting shRNA (sh*NR2F1*) (n ≥ 3). (**G**) Cisplatin sensitivity measured by dose–response curve and quantification of the corresponding IC_50_ values in cisplatin-resistant H226/CDDPr cells treated with the NF-κB inhibitor DHMEQ at the indicated concentrations (0, 10, 20, or 50 μg/mL) (n ≥ 3). (**H**) Cisplatin sensitivity measured by dose–response curve and quantification of the corresponding IC_50_ values in cisplatin-resistant PC9/CDDPr cells treated with the NF-κB inhibitor DHMEQ at the indicated concentrations (0, 10, 20, or 50 μg/mL) (n ≥ 3). The data shown in the figure are presented as the mean ± SEM unless otherwise indicated. *p* values were calculated using two-tailed Student’s *t*-test. * *p* < 0.05, ** *p* < 0.01, *** *p* < 0.001. The uncropped blots are shown in File S1.

NF-κB hyperactivation is a known driver of chemoresistance, often through the upregulation of anti-apoptotic genes [15,19,20]. Furthermore, TGFB1 has been shown to activate NF-κB in other cancer contexts to promote a drug-resistant phenotype [18]. To test this link in our model, we examined the NF-κB activity. Immunoblot analysis revealed a consistent increase in the levels of phosphorylated p65 (p-p65), a marker of NF-κB activation, in both H226 and PC9 cisplatin-resistant cells (Figure 1E and Figure 6A,B). Notably, this elevation in p-p65 was inversely correlated with the downregulated expression of both *NAS1* and *NR2F1* in these resistant cells (Figure 1E and Figure 6A,B). We then performed loss-of-function experiments. Strikingly, the specific knockdown of either *NAS1* or *NR2F1* in parental NSCLC cells was sufficient to recapitulate the resistance-associated phenotype, leading to a marked increase in p-p65 levels (Figure 6C–F) and subsequent NF-κB transcriptional activity. To further determine whether NF-κB activation functionally contributes to cisplatin resistance, we treated cisplatin-resistant H226/CDDPr and PC9/CDDPr cells with the NF-κB inhibitor DHMEQ and assessed cisplatin sensitivity. DHMEQ treatment reduced the IC_50_ of cisplatin in both resistant cell lines in a dose-dependent manner, indicating that inhibition of NF-κB partially restored cisplatin sensitivity (Figure 6G,H). These results provide functional evidence that NF-κB activation contributes to the maintenance of the cisplatin-resistant phenotype in NSCLC cells.

Collectively, these findings delineate a comprehensive signaling cascade underlying cisplatin resistance in NSCLC. We proposed a model in which downregulation of *NAS1* impairs the translation of NR2F1. The subsequent loss of NR2F1 relieves its transcriptional repression of *TGFB1*, leading to increased *TGFB1* expression. Upregulated TGFB1 subsequently activates the downstream NF-κB pathway, ultimately driving the acquisition and maintenance of the cisplatin-resistant phenotype.

## 4. Discussion

The development of cisplatin resistance remains a major obstacle in the treatment of NSCLC. It is crucial to improve chemosensitivity and prevent or bypass chemoresistance to enhance the prognosis of NSCLC patients [21]. Therefore, developing novel potential targets related to chemotherapeutic treatment response rates, as well as revealing the underlying mechanisms, is essential for optimizing clinical chemotherapeutical schemes and the treatment of NSCLC. Emerging evidence has highlighted the involvement of *NAS1*, including NSCLC, in tumor development [8], but the specific roles and mechanisms of *NAS1* in cisplatin resistance are poorly understood. By analyzing our RNA sequencing data, we identified lncRNA *NAS1* as the most considerably down-regulated lncRNA in three cisplatin-resistant NSCLC cell lines compared to their corresponding parental cells (Figure 1). Knockdown of *NAS1* further confirmed the negative relationship of *NAS1* and cisplatin resistance of NSCLC (Figure 2). While numerous studies have established that *NAS1* mainly acts as an oncogene across multiple cancer types, tumor-suppressive properties have been documented in specific malignancies, indicating context-dependent regulatory functions that differ among cancer types [8]. In the present study, TCGA database analysis demonstrated significant downregulation of *NAS1* in NSCLC tumors (Figure 1F). However, phenotypic assays following *NAS1* knockdown revealed that although *NAS1* depletion significantly increased cellular chemoresistance, it concomitantly impaired cell migratory capacity (Figure 2I,J). Comparable observations were made in *NR2F1* knockdown experiments (Figure 3K,L), underscoring the multifaceted role of the *NAS1*-*NR2F1* axis in NSCLC. This functional complexity may be attributed to the regulatory effects of the *NAS1*-*NR2F1* axis on cellular dormancy. It has been previously reported that *NAS1* is upregulated in dormant mesenchymal-like breast cancer cells, where it promotes tumor dissemination through translational regulation of *NR2F1*, albeit at the expense of proliferative capacity [9]. Nevertheless, the specific role of the *NAS1*-*NR2F1* axis in modulating cancer phenotypes in NSCLC warrants further investigation.

LncRNAs can modulate gene expression through diverse mechanisms, including functioning as competing endogenous RNAs (ceRNAs) or miRNA sponges, regulating RNA-binding proteins (RBPs), and transcription-dependent activation or repression of neighboring genes [22]. *NR2F1*, located adjacent to *NAS1*, has been previously reported to undergo translational regulation by *NAS1*, which binds *NR2F1* mRNA and recruits the RBP PTBP1 to facilitate internal ribosome entry site (IRES)-mediated translation [9]. Our study confirmed this translational regulatory relationship between *NAS1* and *NR2F1* (Figure 3A). Whether post-translational regulation or altered protein stability contributes to NR2F1 downregulation remains an open question that will be important to address in future studies. The *NAS1*-*NR2F1* axis has been implicated in cellular dormancy regulation, though the underlying mechanisms vary across different cellular contexts: it promotes dormancy in breast cancer cells but drives the transition from dormancy to proliferation in prostate cancer [9,23]. In contrast, we demonstrated that diminished expression of the *NAS1*-*NR2F1* axis enhances cisplatin resistance in NSCLC, with *NR2F1* identified as a crucial downstream mediator of *NAS1* in this process (Figure 4). However, whether dormancy plays a role in cisplatin resistance in NSCLC through the *NAS1-NR2F1* axis requires further experimental validation. Intriguingly, while *NAS1* regulates cisplatin resistance through translational control of *NR2F1*, cisplatin-resistant cell lines exhibited reductions in both NR2F1 protein and *NR2F1* mRNA levels (Figure 3B). This observation implies that NR2F1 downregulation in resistant cells may involve supplementary upstream regulatory mechanisms independent of *NAS1*, underscoring the intricate molecular circuitry underlying cisplatin resistance.

Transforming growth factor-β (TGF-β) is a pleiotropic cytokine implicated in multiple cellular processes—including cell development, proliferation, epithelial–mesenchymal transition (EMT), and immune regulation—via SMAD-dependent and non-SMAD signaling cascades (encompassing PI3K/AKT, MAPK, and NF-κB pathways) [24]. Intriguingly, TGF-β signaling exhibits dichotomous functions in cancer: it serves as a robust tumor suppressor during early tumorigenesis by inducing apoptosis or cell cycle arrest, yet paradoxically facilitates advanced tumor transformation, progression, and metastasis through multidimensional mechanisms [24]. Although TGFβ1 demonstrates context-dependent roles in oncogenesis, accumulating evidence indicates that elevated TGFβ1 expression fosters chemoresistance, with distinct molecular mechanisms identified across various malignancies [16,18,25,26], notably including NF-κB pathway activation-mediated drug resistance [18]. Consistently, our findings reveal that diminished *NAS1*-*NR2F1* axis derepresses *TGFB1* transcription, resulting in its upregulation and subsequent NF-κB pathway activation (evidenced by markedly increased phosphorylated p65 levels) (Figure 5 and Figure 6). These observations were corroborated in cisplatin-resistant cell lines as well as in *NAS1*- or *NR2F1*-deficient cellular models, substantiating the relationship between TGFβ1 overexpression and enhanced chemotherapeutic resistance.

In summary, our findings underscore the pivotal role of the *NAS1*-*NR2F1* axis in modulating cisplatin resistance in NSCLC. Downregulation of this axis derepresses *TGFB1* transcription, leading to NF-κB pathway activation and consequent promotion of cisplatin resistance. NF-κB activation has been widely implicated in cisplatin resistance, in part through promoting pro-survival and anti-apoptotic signaling. While the precise transcriptional regulatory mechanisms through which NR2F1 governs TGFB1 expression warrant further elucidation, therapeutic interventions aimed at restoring *NAS1* expression (such as nucleotide analog administration), suppressing TGFB1 activity, or antagonizing downstream NF-κB signaling—particularly in combination with cisplatin—may offer a promising strategy for overcoming drug resistance in this malignancy. Although this study has validated the proposed mechanism in cell-based assays, the key findings have not yet been confirmed in animal models. Further in vivo and clinical investigations will be necessary to substantiate the translational relevance of this regulatory axis.

## 5. Conclusions

In this study, we report a comprehensive signaling cascade underlying cisplatin resistance in NSCLC. We propose a model in which downregulation of *NAS1* impairs the translation of *NR2F1*. The subsequent loss of NR2F1 relieves its transcriptional repression of *TGFB1*, leading to increased TGFB1 expression. Upregulated TGFB1 subsequently activates the downstream NF-κB pathway, ultimately driving the acquisition and maintenance of the cisplatin-resistant phenotype.

## Data Availability

All data associated with this study are present in the paper or the Supplementary Materials. The raw and processed high-throughput sequencing data in this study are publicly available in GEO (reference number GSE318830).

## Supplementary Materials

The following supporting information can be downloaded at https://www.mdpi.com/article/10.3390/cancers18071159/s1: Figure S1: Validation of candidate lncRNAs differentially expressed in cisplatin-resistant NSCLC; Table S1: List of primers and shRNA used in this study; File S1: Western blots information.
