# Supplementary material for: lncRNA NAS1 Deficiency Drives Cisplatin Resistance via NR2F1-Mediated TGFB1/NF-κB Signaling Axis in NSCLC
Source: Cancers (Basel). 2026 Apr 3;18(7):1159. doi: 10.3390/cancers18071159 (PMC13072194; doi:10.3390/cancers18071159)
Supplement: Supplementary file 1 [file cancers-18-01159-s001.zip › cancers-4193733-supplementary.pdf]

cancers
MDPI

Supplementary Materials: lncRNA *NAS1* deficiency drives cisplatin resistance *via* NR2F1-mediated TGFB1/NF-κB signaling axis in NSCLC

Xianrong Lin, Yuxin Wu, Qi Wu, Wenjun Tao, Jun Zhang and Jun Zhou

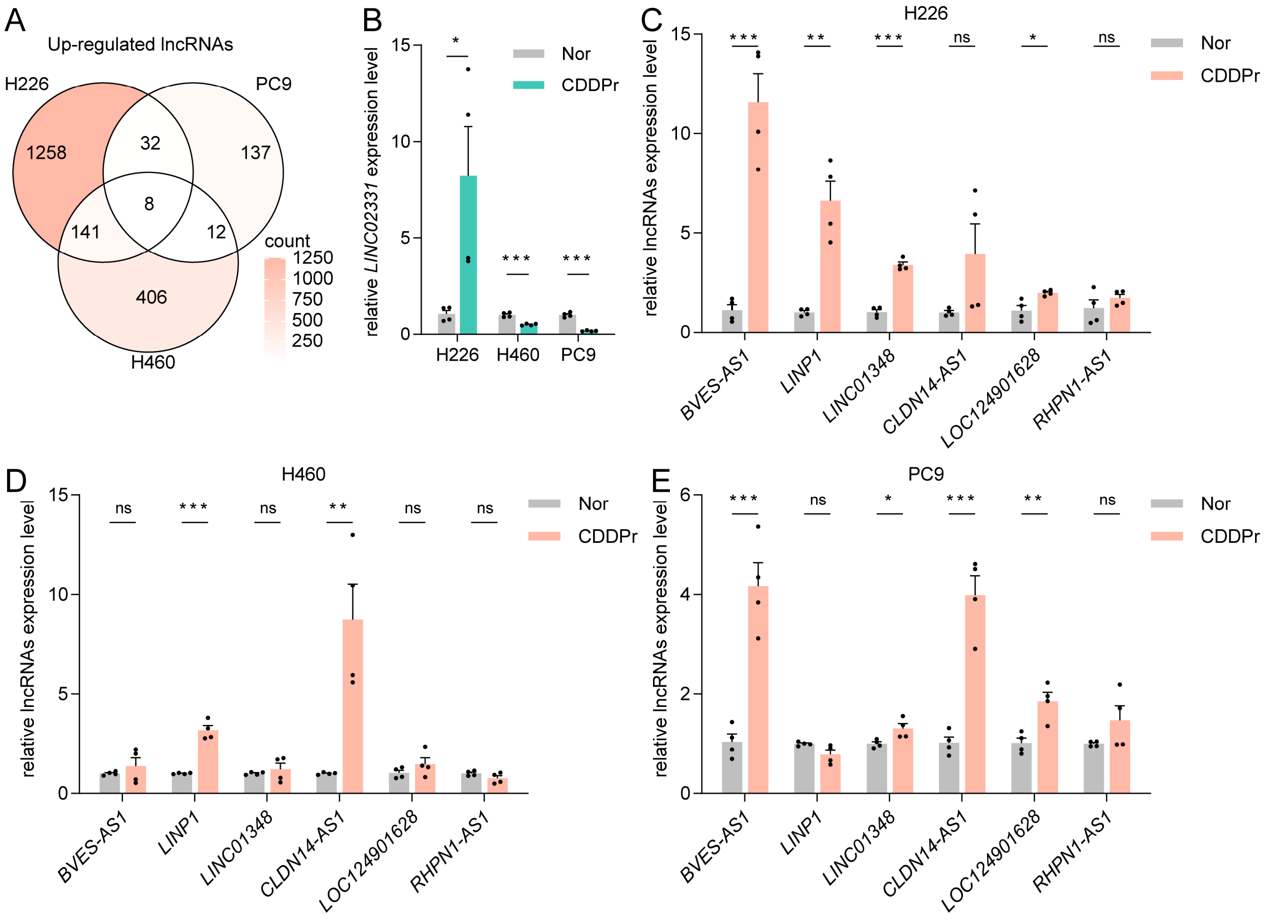

Figure S1. Validation of candidate lncRNAs differentially expressed in cisplatin-resistant NSCLC. (A) Venn diagram of upregulated lncRNAs identified by RNA-seq analysis, showing eight candidates consistently upregulated across all three cisplatin-resistant cell lines (H226/CDDPr, H460/CDDPr, PC9/CDDPr). (B) qPCR validation of LINC02331, one of the two consistently downregulated lncRNAs identified in RNA-seq, in cisplatin-resistant cells compared to parental controls. (C–E) qPCR validation of the eight consistently upregulated lncRNAs in cisplatin-resistant H226 (C), H460 (D), and PC9 (E) cells versus their parental controls. The data shown in the figure are presented as the mean ± SEM unless otherwise indicated. p values were calculated using two-tailed Student's t test. *$p < 0.05$, **$p < 0.01$, ***$p < 0.001$.

Table S1. List of primers and shRNA used in this study.

Primer Name	Sequence (5'-3')	Application
hACTB-qF	ACTCTTCCAGCCTTCCTTCC	qPCR
hACTB-qR	CAATGCCAGGGTACATGGTG	qPCR
NR2F1-qF	TGCCTCAAAGCCATCGTGCTGT	qPCR

NR2F1-qR	CAGCAGCAGTTTGCCAAAACGG	qPCR
qp-TGFB1-F	CAATTCCTGGCGATACCTCAG	qPCR
qp-TGFB1-R	GCACAACTCCGGTGACATCAA	qPCR
hNR2F1-AS1-qF	TTGCCAACAGGCAAATGTCC	qPCR
hNR2F1-AS1-qR	TGGGGAACTGTCATGCCAAA	qPCR
hLINC02331-qF	CTCCTCACAGCAGTGCCTGATTG	qPCR
hLINC02331-qR	AGCCACAACAGTTGAGTGATGACC	qPCR
NR2F1-AS1-633-qF	AGGTGTGGATAAATGAAACT	qPCR
NR2F1-AS1-633-qR	GCATATGGAAATAAGGAAAT	qPCR
hBVES-AS1-qF	GGAGGGACTTCTGACACGC	qPCR
hBVES-AS1-qR	GCTTTGCCTACGATGGAGGG	qPCR
hLINP1-qF	ACTCTGGGTGGGCTGTGTGAC	qPCR
hLINP1-qR	GGAGCTGGCTGAGCAAGTTCTTC	qPCR
hLINC01348-qF	GAGCGTGCCTGTGTCTGATGAG	qPCR
hLINC01348-qR	AGCCCAAAAGGAAGCCGTGATG	qPCR
hCLDN14-AS1-qF	CGGGCTCTGGTTGGTGTTTA	qPCR
hCLDN14-AS1-qR	ACTGCTTTAGGTGGGGTGGA	qPCR
hNALOC124901628-qF	ATGAGACCAGCACGAGAAAG	qPCR
hNALOC124901628-qR	CGGAACATAGGAGAAGGGGA	qPCR
hRHPN1-AS1-qF	GCTCCTGGTCATCAAGTTCCTCT	qPCR
hRHPN1-AS1-qR	GCACAGGCACCAGAATGATCC	qPCR
pcDNA3.1-KOZAK-NR2F1-F	CCAAGCTGGCTAGTTAAGCTTGCCAC-CATGGCAATGGTAGTTAGCAGCTGGCGA-GATCC	Construction of NR2F1 CDS
NR2F1-MYC-1R	GATCCTCTTCAGAGATGAGTTTCTGCTCG-GAGCACTGGATGGACATGTAAGGCCAG-TTG	Construction of NR2F1 CDS
pcDNA3.1-MYC-2R	TGGACTAGTG-GATCCCTACAGATCCTCTTCAGAGATGAG	Construction of NR2F1 CDS
shNR2F1-qF	ACCGGCTCTTCTTCGTCCGTTTGG-TACTCGAGTACCAAACGGACGAAGAA-GAGTTTTTG	shRNA in pRSI9-U6
shNR2F1-qR	CGAACAAAAACTCTTCTTCGTCCGTTTGG-TACTCGAGTACCAAACGGACGAAGAA-GAGC	shRNA in pRSI9-U6
shScramble-F	ACCGGAACAGTCGCGTTT-GCGACTGGCTCGAGCCAGTCGCAAAC-GCGACTGTTTTTTTG	shRNA in pRSI9-U6
shScramble-R	CGAACAAAAAAACAGTCGCGTTTG CGACTGGCTCGAGCCAGTCGCAAAC	shRNA in pRSI9-U6
NC	TTCTCCGAACGTGTCACGT	shRNA in LV3
shNR2F1-AS1-1	GACACTGATATAACTGTAGAT	shRNA in LV3
shNR2F1-AS1-2	GCTGCATCCTTATGGTAGCTA	shRNA in LV3
