# Supplementary material for: lncRNA NAS1 Deficiency Drives Cisplatin Resistance via NR2F1-Mediated TGFB1/NF-κB Signaling Axis in NSCLC
Source: Cancers (Basel). 2026 Apr 3;18(7):1159. doi: 10.3390/cancers18071159 (PMC13072194; doi:10.3390/cancers18071159)
Supplement: Supplementary file 1 [file cancers-18-01159-s001.zip › File S1.pdf]

Figure 3A

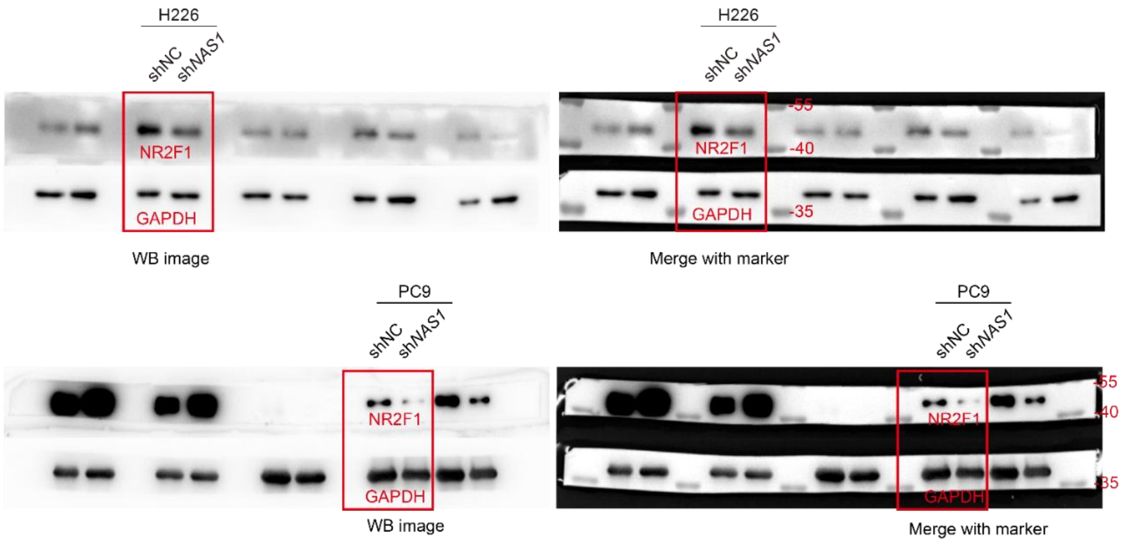

Figure 3C

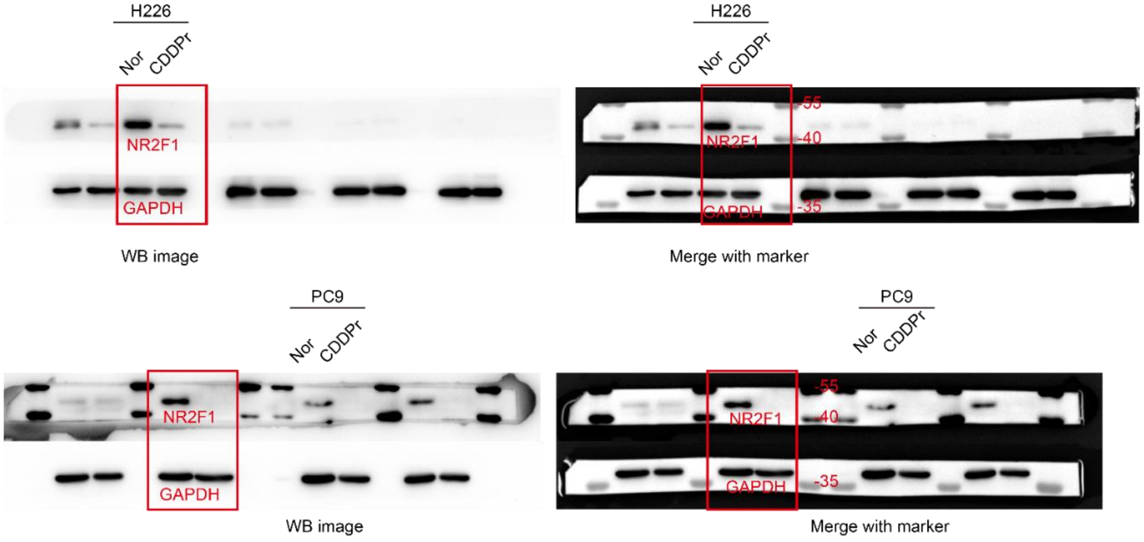

Figure 3E

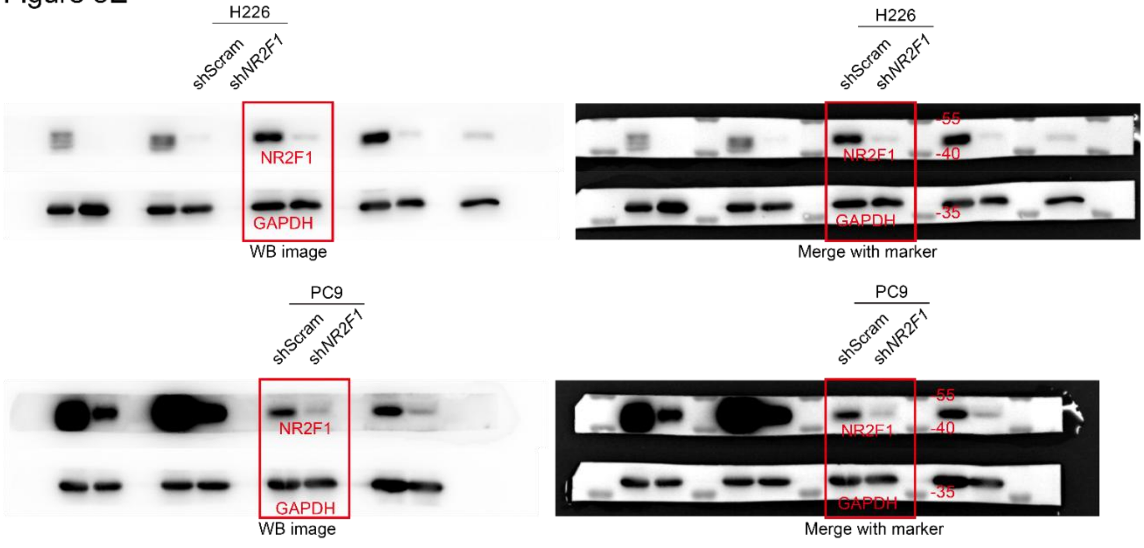

Figure 4A

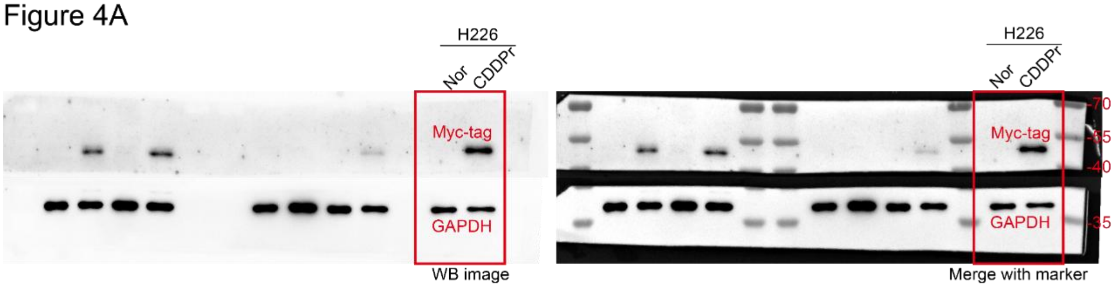

Figure 4B

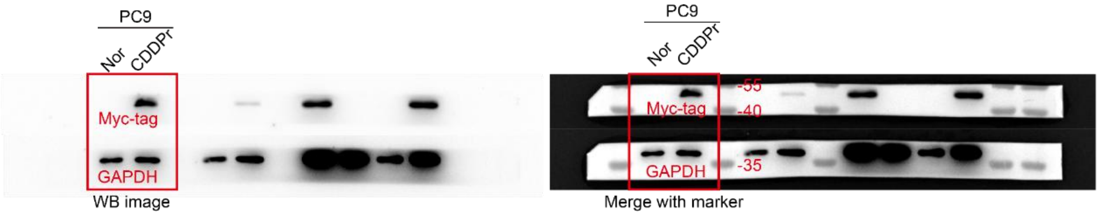

Figure 4G

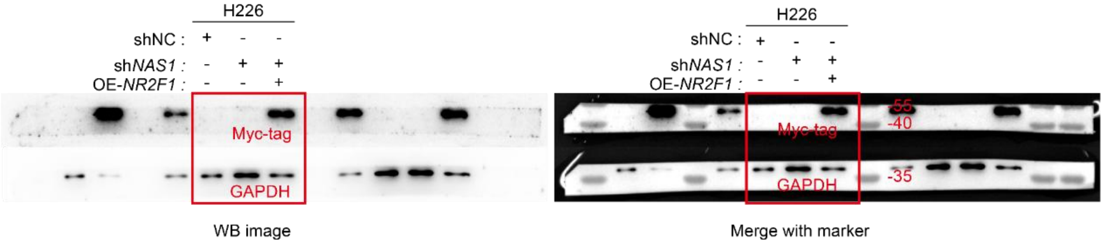

Figure 4H

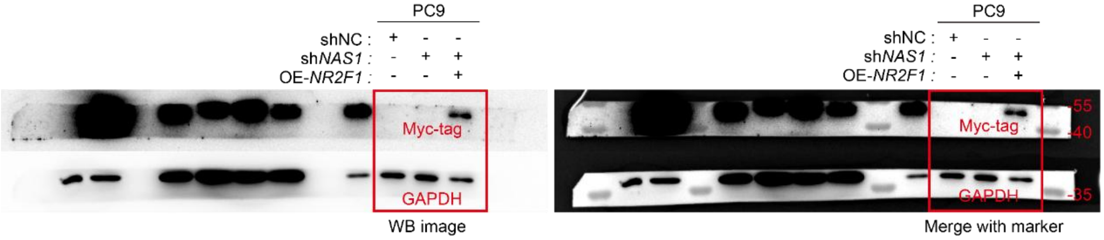

Figure 5H

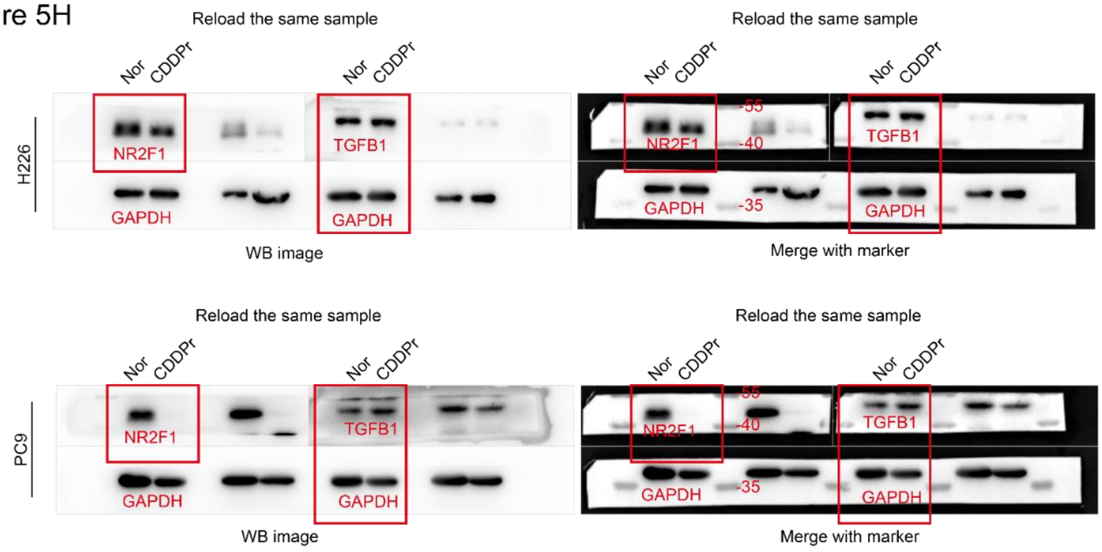

Figure 5I

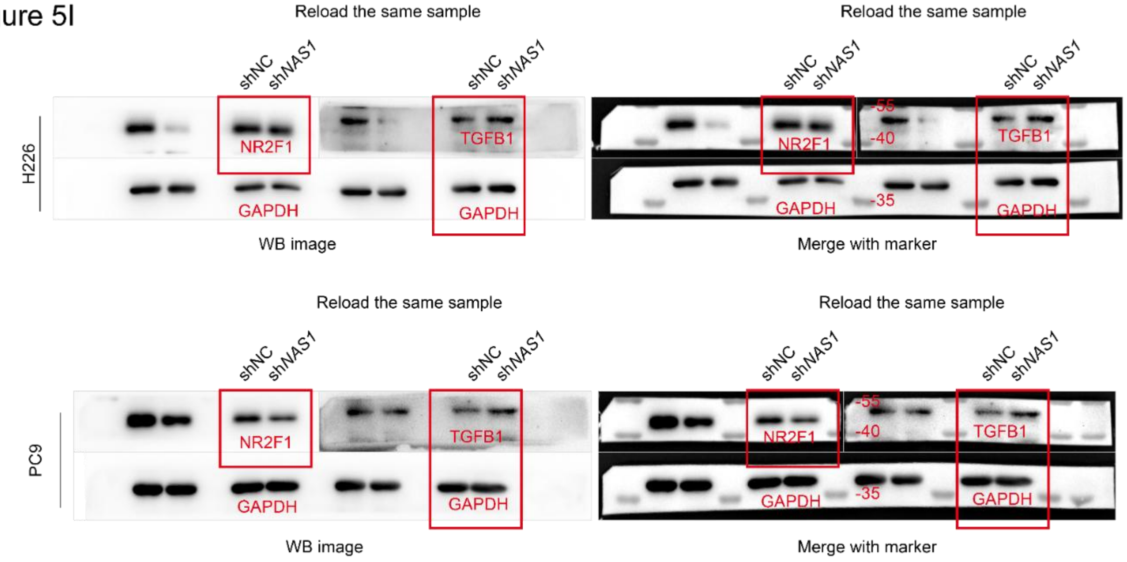

Figure 5J

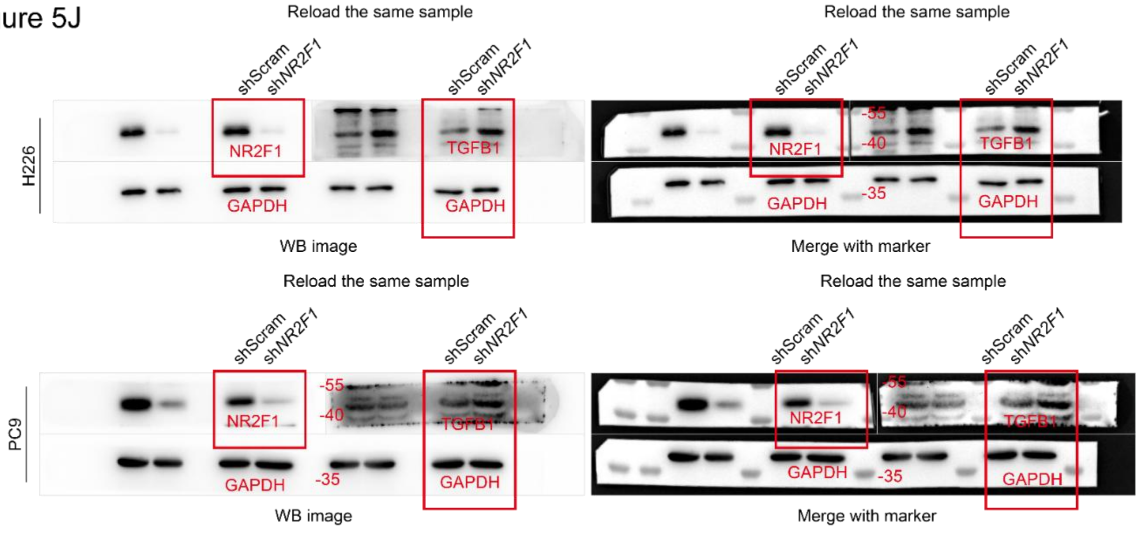

Figure 6A

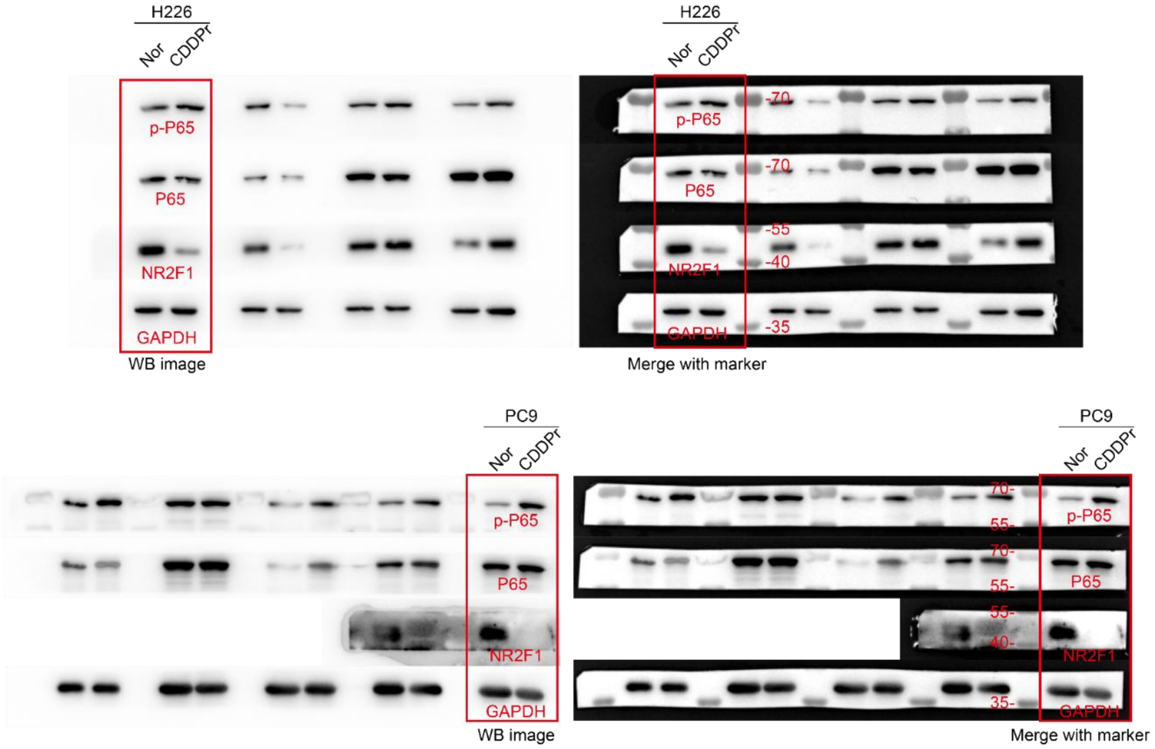

Figure 6C

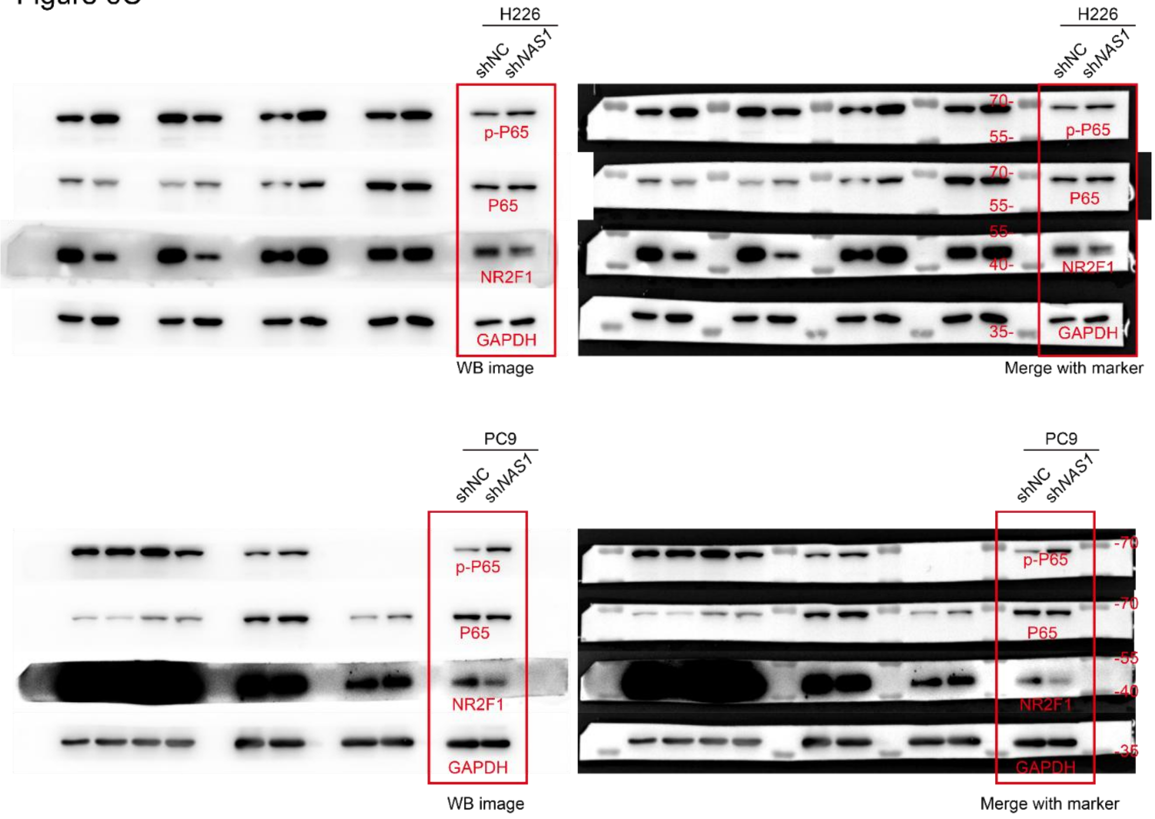

Figure 6E

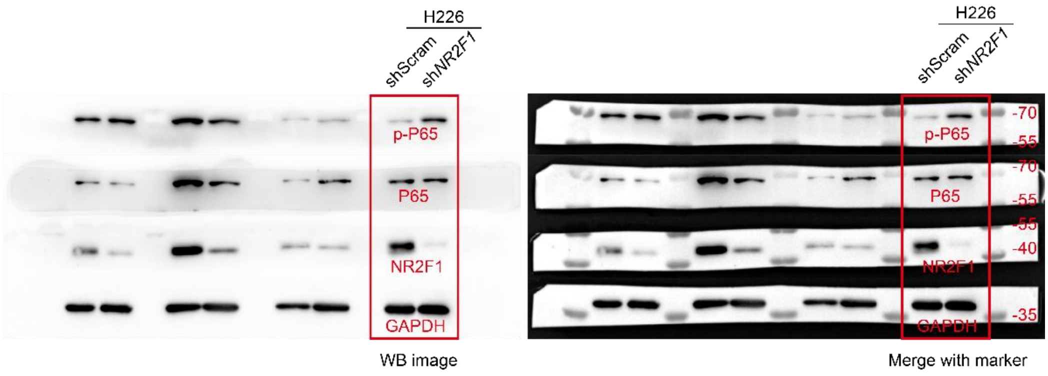
H226
shScram
shNR2F1
p-P65
P65
NR2F1
GAPDH
WB image
H226
shScram
shNR2F1
-70
-55
p-P65
-70
-55
P65
-55
-40
NR2F1
-35
GAPDH
Merge with marker

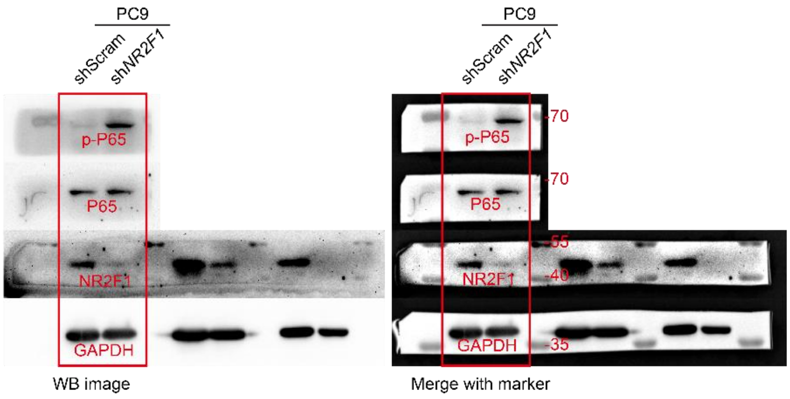
PC9
shScram
shNR2F1
p-P65
P65
NR2F1
GAPDH
WB image
PC9
shScram
shNR2F1
-70
p-P65
-70
P65
-55
-40
NR2F1
-35
GAPDH
Merge with marker
